# Efficacy of Entomopathogenic *Staphylococcus* Bacteria as a Biocontrol Agent against *Rhipicephalus microplus* Ticks: Assessing Reproductive Inhibition and Mortality Rates

**DOI:** 10.3390/microorganisms12030551

**Published:** 2024-03-11

**Authors:** Raquel Cossio-Bayugar, Cesar A. Arreguin-Perez, Hugo Aguilar-Diaz, Estefan Miranda-Miranda

**Affiliations:** 1 Centro Nacional de Investigación Disciplinaria en Salud Animal e Inocuidad, Instituto Nacional de Investigaciones Forestales Agrícolas y Pecuarias INIFAP, Boulevard Cuauhnahuac 8534, Jiutepec 62574, Morelos, Mexico; cesaraarreguinp@gmail.com (C.A.A.-P.); aguilar.hugo@inifap.gob.mx (H.A.-D.); miranda.estefhan@inifap.gob.mx (E.M.-M.); 2 Centro de Investigación en Biotecnología, Universidad Autónoma del Estado de Morelos, Av. Universidad 1001, Cuernavaca 62209, Morelos, Mexico

**Keywords:** *Staphylococcus shinii*, *Staphylococcus succinus*, *Staphylococcu xylosus*, cattle tick, entomopathogenic bacteria

## Abstract

*Rhipicephalus microplus* is a persistent ectoparasite of cattle that causes bovine anaplasmosis and babesiosis, causing economic losses worldwide. Chemical treatment is the primary method for tick control, but the emergence of pesticide-resistant ticks is a major challenge. Alternative biocontrol strategies utilizing entomopathogenic microorganisms are being explored. This study aimed to validate the species identification and assess the efficacy of four strains of *Staphylococcus* bacteria (*S. shinii* S1 and S-2, *S. succinus*, and *S. xylosus*) previously reported as being entomopathogenic to *R. microplus* ticks. According to the bioassays, *S. shinii* S-1 exhibited the greatest degree of reproductive inhibition (47%), followed by *S. succinus* (44.3%) at a concentration of 1 × 10^8^ cfu/mL. *S. xylosus* displayed decreased reproductive inhibition (6.3%). In an additional bioassay, *S. shinii* S-1 exhibited a significant larval mortality of 67.63%, followed by *S. succinus* with 66.75%, *S. shinni* S-2 with 64.61%, and *S. xylosus* with 28.18% mortality. The common signs of infection observed on these ticks included swelling, yellowish exudate on the hypostome, and reduced limb mobility and color change, except for *S. succinus*, which did not cause color changes. These bacteria were naturally found on bovine skin. However, further studies are needed to confirm their potential as promising alternatives or complementary agents to existing acaricidal compounds.

## 1. Introduction

*Rhipicephalus microplus*, commonly known as the cattle tick, is an ectoparasite that infests cattle heavily in tropical and subtropical regions worldwide [1]. These ticks significantly burden their hosts, causing direct harm through blood loss and indirect harm through the transmission of infectious tick-borne diseases [2]. The economic impact of ticks and tick-borne diseases is enormous, with global losses estimated at USD 22–30 billion annually [3]. Specifically, in Latin America, *R. microplus* has a substantial financial impact on the cattle industry, resulting in estimated losses of approximately USD 4.18 billion [4].

The control of tick infestations on livestock typically relies on the application of various acaricides. Unfortunately, the excessive use of acaricides has resulted in the emergence of resistance in *R. microplus*, the primary cattle tick species, relative to the majority of commercially available formulations of these chemical compounds [4]. This development has prompted researchers in the field of tick control to seek alternative methods that do not heavily rely on chemical pesticides. One promising alternative to traditional chemical pesticides is the use of entomopathogenic microorganisms, including bacteria and fungi, as biocontrol agents or tick-specific biopesticides [5,6,7,8,9,10]. These biopesticides can be employed independently or in conjunction with chemical treatments as part of an integrated tick control approach [1,11,12,13].

The most extensively studied entomopathogenic fungi for tick management are *Beauveria bassiana* and *Metarhizium* spp., the latter of which have shown the greatest effectiveness in experimental assays [5,13,14,15]. Among the entomopathogenic bacteria, *Bacillus thuringiensis* and its toxins have been extensively studied as tick-directed biopesticides [16,17]. Other bacteria, including *Proteus mirabilis* [18], *Wolbachia* [19], *Serratia* sp., and *Staphylococcus* sp. [20], have also demonstrated promising effects on cattle ticks. They have demonstrated the ability to induce signs of disease that hinder the completion of a tick’s biological cycle and impede oviposition [20]. These infections can manifest as exudates in the hypostome and genital orifice regions. Further genomic sequencing and phylogenetic analysis of a particular isolate identified it as *S. xylosus*, named INIFAP 005-08 [21]. Furthermore, ticks showing signs of disease were found to be infected with multiple bacteria. Additionally, it was reported that ticks showing similar signs could be infected with multiple bacterial species, with *Staphylococcus* being the most prevalent genus in these tick exudates [22]. Through these studies, novel strains were obtained, and genome sequencing and phylogenetic analysis identified INIFAP 004-15 and INIFAP 009-16 as *S. xylosus*, while strain INIFAP 002-15 was identified as *S. succinus* [21]. The primary objective of this study was to evaluate and describe the bioacaricide activity of these strains against *R. microplus* ticks.

## 2. Materials and Methods

### 2.1. Experimental Animals

Animal care and use were performed according to the Mexican norm NOM-062-ZOO-1999, and the technical specifications for the production, care, and use of laboratory animals can be found at http://www.fmvz.unam.mx/fmvz/principal/archivos/062ZOO.PDF, accessed on 8 February 2024.

### 2.2. Bacterial Strains

Bacterial cultures were obtained from adult ticks exhibiting signs of infection, as previously described by Miranda-Miranda et al. [20]. Bacterial isolates were cryopreserved in 30% glycerol at −70 °C and stored at the Centro Nacional de Investigacion Disciplinaria en Salud Animal e Inocuidad (CENID-SAI-INIFAP) in Jiutepec, Morelos, México. Four bacterial isolates were employed in this study: *Staphylococcus xylosus*, previously identified as INIFAP 005-008 and INIFAP 004-15; INIFAP 002-15, identified as *Staphylococcus succinus* [22]; and *Staphylococcus xylosus*, previously documented as INIFAP-009-16 [21]. All bacterial strains were registered by the World Federation Culture Collection as 1006 (CM-CNRG) and assigned the biosample identifiers SAMN08134550, SAMN08134549, SAMN08134548, and SAMN08134547 as part of the PRJNA421192 bioproject in the GenBank database. To proceed with the experiment, the bacteria were cultured in soy trypticase broth (STA; Sigma—Aldrich, St. Louis, MO, USA) at 37 °C for 16–18 h at 150 rpm. Afterwards, the bacteria were quantified via spectroscopy and set up in 10 mL solutions at different working concentrations (10^6^, 10^7^, 10^8^, and 10^9^ cfu/mL in STA).

### 2.3. Average Nucleotide Identity Comparison

The complete genomes of the *Staphylococcus strains* were previously uploaded to the GenBank with the following accession numbers: GCF_002836835.1, GCF_002836805.1, GCF_002836875.1, and GCF_002836825.1. All genomes were compared to a selected batch of genomes from various *Staphylococcus* species available in the GenBank, including *S*. *shinii* GenBank accession numbers GCA_016774515.1, GCA_001748045.1, GCA_017583065.1GCA_000815285.1, GCA_003041475.1, GCA_003578885.1,GCA_003579155.1, GCA_003578795.1, GCA_003043095.1, *S. xylosus* GenBank accession numbers GCA_020229695.1, GCA_014267365.1, GCA_000706685.1, GCA_002078255.1, GCA_000709415.1, GCA_020229715.1, *S. succinus* GenBank accession numbers GCA_029024945, GCA_001006765.1, GCA_001630745.1, GCA_002902045.1, GCA_014897205.1, and GCA_001902315.1; and *S. saprophyticus* GenBank entry numbers GCA_013341415.1, GCA_007814115.1, GCA_013358365.1, GCA_016067635.1, GCA_000010125.1, and GCA_006094355.1. To perform the comparison, we employed the fastANI tool (v1.34) [23], which utilizes average nucleotide identity (ANI). The default parameters were used for this analysis. Additionally, the resulting data were utilized to create a heatmap using R (4.2.2) and R studio (2022.12.0) [24].

### 2.4. Rhipicephalus (Boophilus) microplus Ticks for Bioassays

Engorged Acaricide-susceptible (Su) Media Joya strain ticks were collected from experimentally infested bovines at the Centro Nacional de Investigacion Disciplinaria en Salud Animal e Inocuidad (CENID-SAI-INIFAP) in Jiutepec Morelos, México, according to a previously reported methodology [25]. The collected ticks were selected for size, with no apparent damage to the capitulum or a darkening color. Approximately 800 engorged female ticks were used in the adult immersion test (AIT) according to a previous method [26], and another group of 120 ticks was used to describe signs of natural infection. Subsequently, the ticks were kept at 28 °C for 15 days, with 80% relative humidity.

### 2.5. Modified Adult Immersion Test (AIT)

AIT [26], with minor modifications was used to evaluate the pathogenic activity of *S. shinii* strain 1 (S-1) (INIFAP 005-008), *S. shinii* strain 2 (S-2) (INIFAP 004-015), *S. xylosus* (INIFAP 009-16), and *S. succinus* (INIFAP 002-2015) against the engorged female *R. microplus* (the bacterial species used are listed in Table 1). Engorged female ticks were randomly assigned to each experimental unit, with four replicates of 10 ticks per experiment. The ticks were exposed to STAs containing 10^6^, 10^7^, 10^8^, or 10^9^ cfu/mL for ten minutes, and the control group was immersed in soy trypticase broth (Sigma—Aldrich). After immersion, the ticks were dried on paper towels and weighed, and each tick was transferred to individual wells in 24-well culture plates. The plates were incubated at 28 °C for 15 days at 80% relative humidity. The percentage of mortality, percentage of morbidity, index of fecundity, and percentage inhibition of oviposition between the different treatments and the control were assessed and compared using the formula provided by the FAO (Supplementary Table S1) [27].

Mortality was determined by counting females showing a lack of motility, including peristalsis; morbidity was determined by counting females with darkening and swelling and those exhibiting hypostome exudates. Egg masses were collected and weighed using an analytical scale, placed in sterile glass vials, and incubated at 28 °C with 80% relative humidity until hatching. The hatching and hatching inhibition rates of each group were observed for 30 days and compared to those of the control treatment group. To estimate the efficacy of the bacterial treatment, the estimated reproduction and the estimated reproduction inhibition were calculated for all groups using the formula provided by the FAO guidelines for chemical acaricide treatment [26,27].

### 2.6. Larval Package Test (LPT)

A previously reported larval package test (LPT) [28,29] was used to measure the entomopathogenic effect on cattle ticks. This test was utilized to assess the pathogenic activity of the bacterial cultures of the *S. shinii* strain 1 (S-1) (INIFAP 005-008), *S. shinii* strain 2 (S-2) (INIFAP 004-015), *S. xylosus* (INIFAP 009-16), and *S. succinus* (INIFAP 002-2015) (the bacterial species used are listed in Table 1).

Approximately 100 larvae were included in the analysis, with three replicates for each treatment. One hundred tick larvae were then transferred to a filter paper package seal on the upper region with adhesive tape, which was impregnated with 2 mL of STA containing 10^8^ cfu/mL of each bacterial strain *(S*. *shinii* S-1, *S. shinii* S-2, *S. xylosus*, and *S. succinus*). At the same time, the control group received soy trypticase broth. The larvae were exposed to these conditions for 48 h at a temperature of 28 °C and a relative humidity of 80%. After the 48 h period, the surviving larvae were counted. Live larvae were identified as those that moved upward when the package was placed vertically and stuck to the upper region on the adhesive tape. Conversely, larvae that did not move were considered dead. The mortality rate was then calculated using the formula in Table S1.

### 2.7. Description of Signs of Infection

The experimental setup consisted of four replicates for each tick treatment group, along with a control group (comprising *S. shinii* S-1, *S. shinii* S-2, *S. xylosus*, and *S. succinus*) consisting of six engorged female ticks per experimental unit. A total of 120 acaricide-susceptible (Su) ticks from the Media Joya strain were used in this study. Before the experiment, the female ticks were washed twice with 100 mL of 10% benzal and then rinsed with 100 mL of distilled water. The ticks were divided into four groups and submerged in a 10 mL bacterial suspension of 1 × 10^8^ cfu/mL in fresh culture media (STA) for 10 min. An additional control group was submerged only in fresh culture media. After treatment, the ticks were dried using paper towels and weighed. Subsequently, each tick was transferred individually to a 24-well culture plate. The plates were further incubated at 28 °C for 15 days at 80% relative humidity to document changes in color, egg darkness, swelling, hemorrhagic lesions (reddening), and hypostome exudates, thereby enabling the assessment of infection.

### 2.8. Isolation of Staphylococcus Bacteria from Bovine Skin

Healthy female Holstein bovines (one year old, housed, and free from previous tick infection) were swab-screened to determine the presence *of Staphylococcus* bacteria on their skin. Samples from different bovine body parts, such as the back, ears, armpit, groin, and perineal region, were taken using sterile swabs moistened with phosphate-buffered saline (PBS). The samples were cultured on *Staphylococcus* 110 agar (MCD LAB) and incubated for 12 h at 37 °C. Any colonies with the appropriate phenotype (color, size, consistency, and colony morphology) were isolated and grown in liquid culture medium with constant agitation for 24 h; the bacteria were subsequently centrifuged, and the resulting bacterial pellets were processed for DNA extraction using the Promega Wizard^®^ Genomic DNA Purification Kit. Colony PCR was conducted with a specific pair of oligos for the chaperonin dnaJ/hsp40: GCCAAAAGAGACTATTATGA and ATTGYTTACCYGTTTGTGTACC [30]. The PCR procedure began with an initial denaturation temperature of 94 °C for 5 min; 35 amplification cycles of 94 °C denaturation for 45 s, 50 °C alignment for 30 s, and 72 °C extension for 60 s; and a final extension of 72 °C for 10 min. PCR products were sequenced at the “Unidad de Síntesis y secuenciación de ADN del Instituto de Biotecnología” in both directions (3′–5′ and 5′–3′) using the same oligonucleotides used for amplification.

### 2.9. Statistical Analyses

The effects of bacterial concentrations (10^6^, 10^7^, 10^8^, and 10^9^) on adult female tick mortality, morbidity, percentage inhibition of oviposition, larval hatching inhibition, and larval mortality were analyzed using one-way ANOVA. Post hoc tests were conducted using the Tukey test (*p* < 0.05). Statistical analyses were performed using R and R Studio software [24].

Nonparametric tests were employed to analyze the signs of infection since the data did not follow a normal distribution, as confirmed by the Shapiro—Wilk test. Specifically, the Kruskal—Wallis and Dunn tests were used to compare the data. Statistical analyses were conducted using R software version 4.2.2 with the assistance of R Studio software (2022.12.0) [24].

## 3. Results

### 3.1. Genomic Comparison of Average Nucleotide Identity

**Figure 1:**
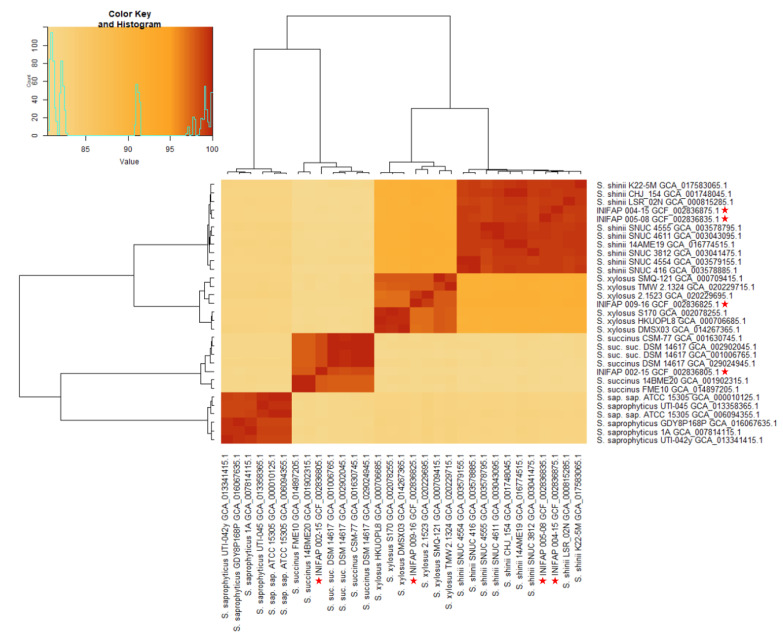
Average nucleotide identity (ANI) heatmap depicting the distribution of bacterial genomes in our dataset with our four genomes indicated by a red star. The heatmap includes genome information obtained from the NCBI genome database, and the species are categorized based on a >95% ANI cutoff. S. sap. sap. refers to *S. saprophyticus* subsp. *saprophyticus*, and *S.* suc. *suc*. corresponds to *S. succinus* subsp. *succinus.* Stars indicate the *Staphylococcus* strains used in this work. Darker rectangles represent higher identity.

In our previous research, we extracted bacteria from infected ticks and identified INIFAP 004-15, INIFAP 004-15, and INIFAP 009-15 as *S. xylosus* and INIFAP 002-15 as *S. succinus* [21]. To corroborate these initial identifications, we conducted average nucleotide identity (ANI) analysis to evaluate the genomic similarity at the nucleotide level among various strains. Table 1 presents an overview of the most notable ANI-based matches discovered between our and reference genomes. The ANI comparison heatmap (Figure 1) provides a clear visual representation of the presence of four closely related groups, each with a similarity of more than 95%. Specifically, there is coherence in the genome between INIFAP 004-15 and INIFAP 005-08, indicating their close relationship with *S. shinii*. Similarly, INIFAP 002-15 is closely related to *S. succinus*, while INIFAP 009-15 is similar to *S. xylosus*. Therefore, to avoid confusion, we named INIFAP 004-15 and INIFAP 005-08 *S.* as *shinii* S-1 and *S. shinii* S-2, respectively.

### 3.2. Adult Immersion Test (AIT)

**Figure 2:**
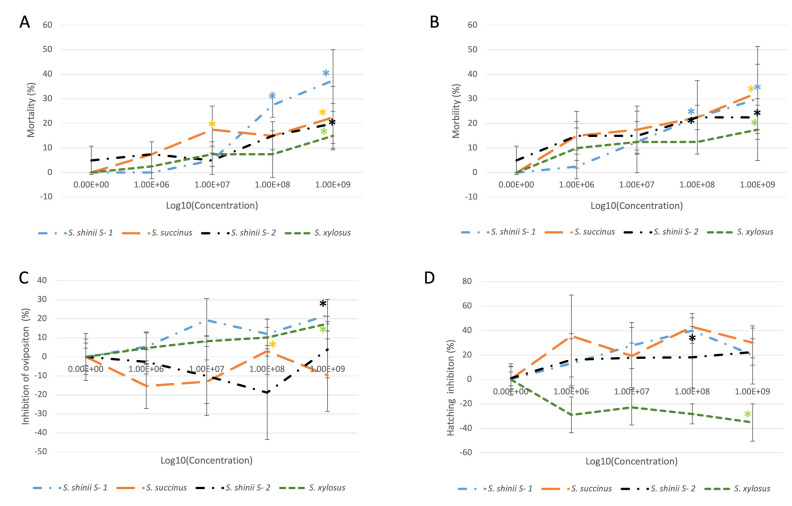
Effects of bacterial concentration on parameters of the Adult Immersion Test. (**A**) Mortality (%), (**B**) morbidity (%), (**C**) inhibition of oviposition (%), and (**D**) hatching inhibition (%). The four bacterial strains are represented by different colors: *S. shinii* S-1 (blue), *S. succinus* (orange), *S. shinii* S-2 (black), and *S. xylosus* (green). * Indicates statistically significant differences.

*S. shinii* S-1 exhibited the greatest mortality level of engorged ticks (37%), followed by *S. succinus* and *S. shinii* S-2 (22.5 and 20% mortality, respectively) at a bacterial concentration of 1 × 10^9^ cfu/mL (Figure 2, Supplementary Tables S1–S3). The percentage inhibition of oviposition was the highest for *S. shinii* S-1 (21.9%), followed by *S. xylosus* (17.4%) at a 1 × 10^9^ cfu/mL concentration. The hatching percentage showed that *S. succinus* (43%) had the highest degree of hatching inhibition, followed by *S. shinii* S-1 (39.9%) at 1 × 10^8^ cfu/mL concentration. Contrary to the anticipated findings, it is worth mentioning that both strains displayed decreased hatching inhibition at a higher concentration of 1 × 10^9^ cfu/mL. Specifically, *S. succinus* decreased by 29.9%, while *S. shinii* S-1 decreased by 21.2% (Figure 2, Supplementary Tables S2–S5).

**Figure 3:**
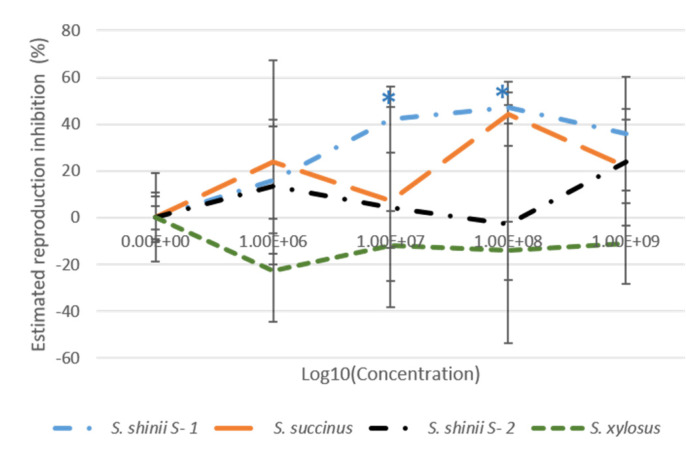
Effects of bacterial concentration on estimated reproduction inhibition of the Adult Immersion Test. The four bacterial strains are represented by different colors: *S. shinii* S-1 (blue), *S. succinus* (orange), *S. shinii* S-2 (black), and *S. xylosus* (green). * Indicates statistically significant differences.

With respect to the estimated reproduction inhibition, *S. shinii* S-1 achieved the highest value (47%), followed by *S. succinus* (44.3%) at a concentration of 1 × 10^8^ cfu/mL. However, these values decreased at a higher concentration of 1 × 10^9^ cfu/mL, 35.8% for *S. shinii* S-1 and 21.66% for. *S. succinus.* Only the reproduction inhibition results for *S. shinii* S-1 were statistically significant (Figure 3, Supplementary Tables S2 and S4).

*S. shinii* S-1 significantly affected all parameters, achieving the best global results at a concentration of 1 × 10^8^ cfu/mL. These included 27.5% mortality, 22.5% morbidity, 12% inhibition of oviposition, 39.9% hatching inhibition, and an estimated reproduction inhibition of 47% (Figure 2 and Figure 3, Supplementary Table S2).

Similarly, at the higher concentration (1 × 10^9^ cfu/mL), there were noteworthy results: a significant mortality rate of 37.5%, a morbidity rate of 30%, a percentage of inhibition of oviposition of 21.9%, hatching inhibition of 21.2%, and an overall estimated reproduction inhibition of 35.8% (Figure 2 and Figure 3, Supplementary Table S2).

### 3.3. Larval Package Test (LPT)

We performed a modified version of the larval package test at 1 × 10^8^ cfu/mL for each strain. The different *Staphylococcus* strains caused significant mortality in the *R. microplus* larvae (Table 2).

### 3.4. Description of Signs of Infection

**Figure 4:**
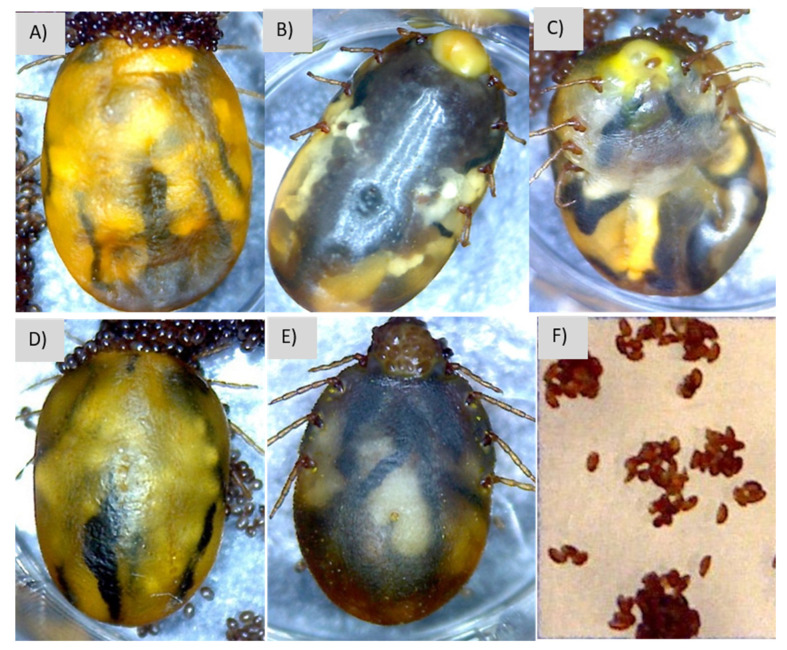
*R microplus* females in the control group (**A**), *S. shinii* S-I (**B**), *S. shinii* S-2 (**C**), *S. succinus* (**D**), *S. xylosus* (**E**), and malformed eggs from *S. shinii* S-I (**F**).

Signs of infection included darkening, swelling, hypostome exudates, darker eggs, swelling, reduced oviposition, or dry eggs. Specifically, the infection caused by *S. shinii* S-I and S-2 resulted in exudate and swelling (Figure 4B,C, Table 3). *S. shinii* S-1 infections resulted in dried eggs, although the differences were not significant (Figure 3F). On the other hand, the infection caused by *S. succinus* resulted in exudate and significant differences in swelling and limb mobility (Figure 4D, Table 3). Finally, *S. xylosus* led to swelling and statistically significant differences in exudate (Figure 4E, Table 3). All strains exhibited slight color changes, except for *S. succinus*, where no color change was evident.

### 3.5. Isolation of Staphylococcus Bacteria on Bovine Skin

**Figure 5:**
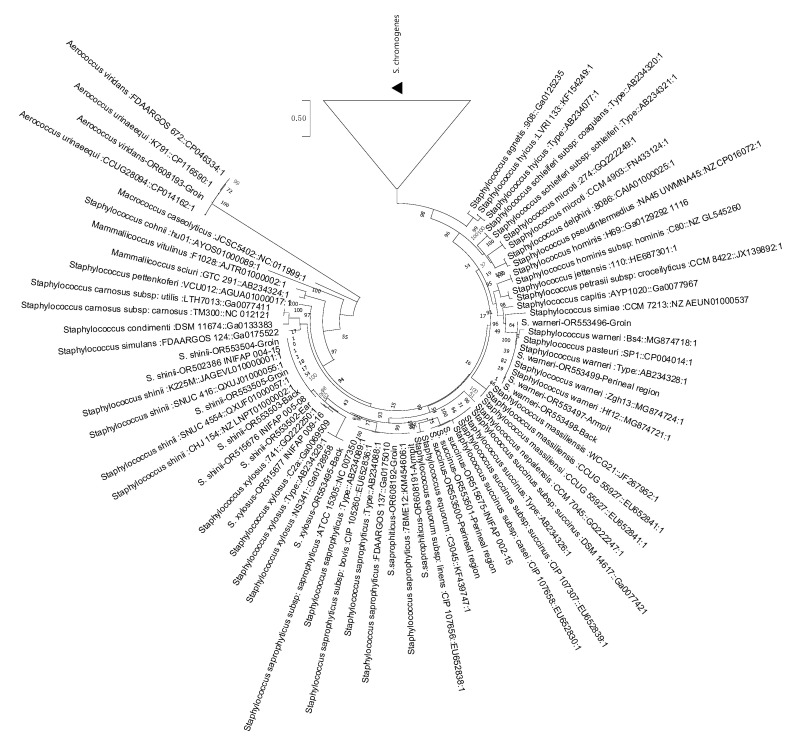
Phylogenetic inference of bacterial strains from bovine skin based on the dnaJ/hsp40 gene. The analysis was performed using the maximum likelihood method and the general time-reversible model on the MEGA X platform. The root species used for the analysis was *Macrococcus caseolyticus*, and the resulting tree was validated through bootstrapping with 1000 repetitions. Only boostrap values above 80 are shown. The numbers on the termini indicate the corresponding samples obtained from bovine skin.

To confirm the presence of *Staphylococcus* on bovine skin and gain further insights into its natural population and interaction with ticks, samples were collected from various regions of the skin of two bovines. These regions included the groin, back, armpit, peritoneum, and ear, which are known to harbor ticks during infestations. Through phylogenetic inference, we identified six *Staphylococcus* species: *S. shinni*, *S. chromogenes*, *S. pasteuri*, *S. succinus*, *S. xylosus*, *S. saprophyticus*, and *Aerococcus* spp. (Figure 5, Table 4). Additionally, Table 3 shows the distribution of these *Staphylococcus* species on bovine skin. *S. shinni* was found on the ear, back, and groin regions, while *S. xylosus* was found on the animal’s back. Finally, *S. succinus* was present in the perineal area. The most prevalent bacterium, *S. chromogenes*, was primarily found in the ear, followed by the back, groin, and armpit.

## 4. Discussion

Previous studies elucidated the presence of the entomopathogenic *Staphylococcus saprophyticus* responsible for inducing infectious diseases in cattle ticks [20]. Follow-up studies reported that ticks exhibiting comparable signs presented multiple bacterial species on tick exudates, with *Staphylococcus* representing the most predominant genus in these tick exudates. The examples of *Staphylococcus* species identified in these exudates include *S. succinus* and *S. xylosus* [22]. Based on the phylogenomic ANI results, it can be firmly concluded that the strains INIFAP 005-008 and INIFAP 004-15 [20,21,22] belong to *Staphylococcus shinii,* although they were previously identified as *S. xylosus* via a less rigorous molecular taxonomic analysis. Notably, *S. shinii* is a newly discovered species that was previously isolated from fresh vegetables and was not available for comparative genomics analysis; it is described as coagulase-negative and classified as a methicillin-resistant organism phylogenetically related to *Staphylococcus pseudoxylosus* [31,32].

*Staphylococcus xylosus* and *S. succinus* are commonly regarded as normal commensals of farm animals, including bovine skin [33,34], and they are commonly found on ticks [35,36,37]. However, certain strains of these bacteria have acquired the ability to infect ticks and impact their viability, as demonstrated in previous research [20]. Therefore, it is important to investigate how these bacteria become infectious to ticks. Furthermore, it is worth exploring whether different strains of these bacteria may exert varying effects on tick populations.

This study focused on the assessment of the bioacaricidal effects of three *Staphylococcus* strains, *S. shinni* S-1, *S. shinni* S-2, and *S. succinus*, which were isolated from infected tick exudates, along with one strain of *S. xylosus* isolated from the hemolymph of an infected tick [20,21,22]. Bioassays were also conducted to assess the pathogenic impact of these bacterial strains on ticks. Notably, during the adult immersion test at a concentration of 1 × 10^8^ cfu/mL, *S. shinni* S-1 exhibited reproductive inhibition at 47%, which was accompanied by 27.5% mortality, 22.5% morbidity, 12% oviposition inhibition, and 39.9% hatching inhibition. It is important to mention that the AIT treatment of *S. shinni* S-2 did not significantly affect these parameters, except for a 22.5% observed morbidity (Figure 2 and Figure 3, Tables S1 and S2). Additionally, the genome analysis conducted previously revealed a discrepancy in genome size between the two *S. shinni* strains, with *S. shinni* S-1 having a genome size of 3.07 Mbp (3070102 bp) compared to 2.9 Mbp (2982247 bp) for *S. shinni* S-2. A more comprehensive genomic analysis is necessary to identify the specific genes that are absent in the smaller genome strain and assess their potential role in the entomopathogenic effect on the cattle tick.

*S. succinus* had significant effects on AIT, with a reproductive inhibition of 44.3% at a concentration of 1 × 10^8^ cfu/mL (Figure 2 and Figure 3, Table S3). In comparison, *S. xylosus* exhibited a lower reproductive inhibition of 6.3% (Figure 2 and Figure 3, Table S4). This strain has a lower acarapathogenic ability.

Further research is required to explore the potential enhancement of reproductive inhibition by modifying culture media conditions, bacterial combinations, and exposure times since the bacteria must infect the tick to manifest their acaropathogenic activity. During this study, we evaluated the end of the tick’s life cycle, and we are aware that it is crucial to assess the prepatent period for infection or exposure during earlier tick stages to accurately evaluate the potential of these bacteria as acaropathogenic agents. However, a preliminary analysis was designed to ascertain the acarapathogenic effects of these bacteria on ticks; we realize that additional studies involving animals are essential for determining the genuine biocontrol potential of these bacteria.

The optimal entomopathogenic effect, as measured by the inhibition of the reproduction rate, was observed at a concentration of 1 × 10^8^ cfu/mL. This concentration encompasses the combined effects of mortality, oviposition, and hatching. On the other hand, lower values were observed at a concentration of 1 × 10^9^ cfu/mL. These results highlight the significance of a high bacterial concentration in determining the pathogenic abilities of *S. shinni* S-1 and *S. succinus*. However, it is worth noting that previous studies have suggested that *Staphylococcus aureus* can enter a dormant phenotypic state as a survival strategy with increasing cell density [38]. It remains to be investigated whether this phenomenon also influences the acaropathogenic capacities of these strains. Therefore, further research is necessary to explore the potential impact of higher bacterial concentrations on the virulence and acaropathogenic abilities of *Staphylococcus* bacteria.

A significant effect on larval mortality was observed across all strains tested in our study. Notably, *S. shinii* S-1 and S-2 and *S. succinus* exhibited high larval mortality rates exceeding 64%. In comparison, *S. xylosus* exhibited a lower but significant mortality rate of 28.18%. Moreover, both *S. shinnii* and *S. succinus* demonstrated significantly greater acaropathogenic effects than *S. xylosus* (Table 2). These findings indicate that all three *Staphylococcus* species can infect and induce an infectious disease that impacts the viability of *R. microplus* larvae. Further experiments should be conducted to investigate the potential synergistic effects of combining different bacteria on tick viability, which could lead to improved results.

Our study aimed to identify specific pathogenic effects associated with different *Staphylococcus* species found in infected cattle ticks. These infections present with various signs, including the presence of exudates in the hypostome area and genital orifice, as well as darkening, swelling, reduced limb mobility, and darker or drier eggs [20]. The results of our study revealed that all *Staphylococcus* species tested in the bioassays could induce signs of infection. Notably, *S. xylosus* had the most pronounced effect on exudate production, while *S. succinus* had significant effects on swelling and limb mobility (Table 3).

Ticks are believed to acquire bacteria that cause infections when they feed on the blood of bovines, as these bacteria have been found on the skin microbiota of cattle [20]. To further investigate this phenomenon, we analyzed the natural *Staphylococcus* microbiota present on the skin of cattle with no previous tick infection. Our study successfully confirmed the presence of *Staphylococcus* species in cattle that can induce infection in ticks, indicating that ticks can acquire these infections from their bovine hosts. Notably, phylogenetic analysis revealed that most of these bacteria (61.1%) were closely related to *S. chromogenes*. The second most abundant species identified were *S. shinii* (11.1%) and *S. warneri* (11.1%). Importantly, this study is the first to report the presence of *S. shinii* on bovine skin. Research revealed that *S. shinii* was detected in multiple sampling areas, such as the groin, back, and ears, which aligns with the typical locations where ticks are commonly found on bovine bodies [39]. In contrast, *S. xylosus* was predominantly observed on the animal’s back. Interestingly, it was noted that *S. succinus*, which was originally isolated from the perineum, was also present in the same region. Further experiments will be required to assess the prevalence of these bacteria in the normal microbiota by examining bovines from different breeds and geographical regions.

This study highlighted the potential of *Staphylococcus* species, including *S. shinii*, *S. xylosus*, and *S. succinus*, as natural biocontrol agents against ticks. These bacteria have demonstrated promising results in laboratory settings, indicating their potential as an alternative or complementary approach to conventional acaricides for controlling tick populations.

## Figures and Tables

**Table 1:** Average nucleotide identity comparison of the four bacterial genomes in our dataset with the best ANI identity.

Strain	Best Average Nucleotide Identity Genome	ANI%	Orthologous Matches	Total Fragments
005-08	*Staphylococcus shinii* strain CHJ_154 GCA_001748045.1	99.21	894	1012
004-15	*Staphylococcus shinii* strain K22-5 M GCA_017583065.1	99.21	913	979
002-15	*Staphylococcus succinus* strain DSM 14617 GCA_029024945.1	98.60	860	891
009-16	*Staphylococcus xylosus* strain 2.1523 GCA_020229695.1	99.07	879	919

**Table 2:** Mortality of *R. microplus* larvae according to treatment. Superscript denotes statistically significant differences.

Treatment	Mortality (SD)
Control	19.7(±0.35) ^a^
*S*. *shinii* S-1	67.63(±1.79) ^b^
*S. succinus*	66.75(±14.16) ^b^
*S*. *shinii* S-2	64.61(±2.11) ^b^
*S. xylosus*	28.18(±7.05) ^ab^

**Table 3:** Medians and ranges of signs according to treatment superscripts denote statistically significant differences. Each median is shown with the corresponding quartile range in parentheses.

Treatment	Swelling	Color Change	Exudate	Limb Mobility	Dried Eggs
Control	0 (0–0) a	0 (0–0) NS	0 (0–0) a	0 (0–0) a	0 (0–0) NS
*S. shinii* S-1	37.5 (25–75) ab	0 (0–25) NS	50 (25–75) ab	25 (25–25) ab	0 (0–25) NS
*S. shinii* S-2	50 (0–100) ab	25 (0–50) NS	50 (25–100) ab	25 (0–25) ab	0 (0–0) NS
*S. succinus*	87.5 (75–100) b	0 (0–0) NS	50 (0–75) ab	62.5 (0–75) b	0 (0–0) NS
*S. xylosus*	62.5 (25–100) ab	0 (0–25) NS	87.5 (25–100) b	25 (0–50) ab	0 (0–0) NS

Letters indicate statistically significant differences (*p* < 0.05), and NS indicates no significant difference.

**Table 4:** Distribution of *Staphylococcus* species on bovine skin. The colony counts and percentage composition by species are shown in parentheses.

	Armpit	Groin	Perineal Region	Back	Ears	Total
*S. chromogenes*	1 (33.3%)	2 (29%)	1 (25%)	3 (50%)	15 (94%)	22 (61.1%)
*S. shinii*	0	2 (29%)	0	1 (16.6%)	1 (6%)	4 (11.1%)
*S. warneri*	1 (33.3%)	1 (14%)	1 (25%)	1 (16.6%)	0	4 (11.1%)
*S. saprophyticus*	1 (33.3%)	1 (14%)	0	0	0	2 (5.5%)
*S. succinus*	0	0	2 (50%)	0	0	2 (5.5%)
*S. xylosus*	0	0	0	1(16.6%)	0	1 (2.7%)
*Aerococcus* spp.	0	1 (14%)	0	0	0	1 (2.7%)

## Data Availability

Data are contained within the article, and links to public databases are described.

## Supplementary Materials

The following supporting information can be downloaded at https://www.mdpi.com/article/10.3390/microorganisms12030551/s1. Table S1: Formulas used to calculate parameters in the bioassays; Table S2: *S. shinni* S-1 (INIFAP 005-08) bioacaricidal activity of several variables obtained via modified AIT against the susceptible (Media Joya) *R. microplus* strain; Table S3. *S. shinni* S-2 (INIFAP 004-15) bioacaricidal activity of several variables obtained via modified AIT against the susceptible (Media Joya) *R. microplus* strain; Table S4. *S. succinus* (INIFAP 002-15) bioacaricidal activity of several variables obtained via modified AIT against the susceptible (Media Joya) *R. microplus strain;* Table S5. *S. xylosus* (INIFAP 009-16) bioacaricidal activity of several variables obtained via modified AIT against the susceptible (Media Joya) *R. microplus* strain.
