# Supplementary material for: Efficacy of Entomopathogenic Staphylococcus Bacteria as a Biocontrol Agent against Rhipicephalus microplus Ticks: Assessing Reproductive Inhibition and Mortality Rates
Source: Microorganisms. 2024 Mar 11;12(3):551. doi: 10.3390/microorganisms12030551 (PMC10974063; doi:10.3390/microorganisms12030551)
Supplement: Supplementary file 1 [file microorganisms-12-00551-s001.zip › Table S1 Formulas .pdf]

Table S1. Formulas Used to Calculate Parameters in the Bioassays.

Modified Adult Immersion Test (AIT)	
**Parameter**	**Formula**
Mortality (%)	Mortality = (Dead females / Total females) x 100
Morbidity (%)	Morbidity = (Sick females / Total females) x 100
Index of fertility (IF%)	IF% = (Egg mass / Female initial mass) x 100
Percentage inhibition of oviposition (%OI)	OI% = ((EPI% Control – EPI% Treatment) / EPI% Control) x 100
Hatching (H%)	H% = (Hatched larvae / (Hatched larvae + Unhatched eggs)) x 100
Hatching Inhibition (HI%)	HI% = ((EC% Control – EC% Treatment) / EC% Control) x 100
Estimated Reproduction (ER)	ER = (EPI% / 100) x (EC% / 100) x 20000
Estimated Reproduction Inhibition (ERI)	ERI = ((RE Control – RE Treatment) / RE Control) x 100
Larval Package Test (LPT)	
Mortality (%)	Mortality = (Dead larvae / Total larvae) x 100
