# Supplementary material for: Efficacy of Entomopathogenic Staphylococcus Bacteria as a Biocontrol Agent against Rhipicephalus microplus Ticks: Assessing Reproductive Inhibition and Mortality Rates
Source: Microorganisms. 2024 Mar 11;12(3):551. doi: 10.3390/microorganisms12030551 (PMC10974063; doi:10.3390/microorganisms12030551)
Supplement: Supplementary file 1 [file microorganisms-12-00551-s001.zip › Table S2 corrected version.pdf]

Table S2. *S. shinni* S-1 (INIFAP 005-08) bioacaricidal activity of several variables obtained by modified AIT against the susceptible (Media Joya) *R. microplus* strain.

Variable	Control	1x10^6	1x10^7	1x10^8	1x10^9
Mortality (%)	(± 0)a	0 (± 0)a	5 (±5.8)a	27.5 (± 5)b	37.5 (± 12.6)b
Morbidity (%)	0 (± 0)a	2.5 (±5)a	12.5 (±12.6)ab	22.5 (±5)b	30 (±14.1)b
Index of fertility (IF%)	41.7 (±5.1)a	39.5 (±3)ab	33.7 (±4.1)ab	36.7 (±3.4)ab	32.5 (±3.4)b
Percentage inhibition of oviposition (%OI)	0 (±12.3)a	5.3 (±7.3)ab	19.3 (±11.5)ab	12.0 (±7.9)ab	21.9 (±8.3)b
Hatching (H%)	54.5(±5.6)a	47.6(±11) ab	39.6 (±10.3)ab	33 (±5.7) b	43.8(±13.0)ab
Hatching Inhibition (HI%)	0 (±10.2)a	13.3 (±20)ab	27.8 (±18.9)ab	39.9 (±10.5)b	21.2 (±23.8)ab
Estimated Reproduction (ER)	4520.7 (±477.9)a	3790.6(±1040.3)ab	2620.8 (±638.6)b	2396.4 (±306.4)b	2902.3 (±1096.2)ab
Estimated Reproduction Inhibition (ERI)	0 (±10.6)a	16.2 (±23.0)ab	42 (±14.1)b	47 (±6.8)b	35.8 (±24.3)ab

Letters indicate statistically significant differences (P < 0.05), and NS indicates no significant difference.
