# Supplementary material for: Efficacy of Entomopathogenic Staphylococcus Bacteria as a Biocontrol Agent against Rhipicephalus microplus Ticks: Assessing Reproductive Inhibition and Mortality Rates
Source: Microorganisms. 2024 Mar 11;12(3):551. doi: 10.3390/microorganisms12030551 (PMC10974063; doi:10.3390/microorganisms12030551)
Supplement: Supplementary file 1 [file microorganisms-12-00551-s001.zip › Table S3 corrected version.pdf]

Table S3. *S. shinni* S-2 (INIFAP 004-15) bioacaricidal activity of several variables obtained by modified AIT against the susceptible (Media Joya) *R. microplus* strain.

Variable	Control	1x106	1x107	1x108	1x109
Mortality (%)	5 (± 5.8)a	7.5 (± 5)ab	5 (± 5.8)a	15 (± 5.8)ab	20 (± 8.2)b
Morbidity (%)	5 (± 5.8)a	15 (± 10)ab	15 (± 5.8)ab	22.5 (± 5)b	22.5 (± 5)b
Index of fertility (IF%)	45.7 (±4.3)NS	46.9 (±2.9)NS	50.33 (±9.5)NS	54.3 (±11.3)NS	43.9 (±6.8)NS
Percentage inhibition of oviposition (%OI)	0 (±9.3)NS	-2.6 (±6.4)NS	-10.1 (±20.1)NS	-18.8 (±24.8)NS	3.9 (±14.9)NS
Hatching (H%)	71.1 (±6.7)NS	59.1 (±17)NS	59 (±17.6)NS	58.8(±18.1)NS	55.7 (±7.7)NS
Hatching Inhibition (HI%)	0 (±9.4)NS	16.2 (±21.4)NS	17.9 (±24.5)NS	18.2(±25.2)NS	22.5 (±10.7)NS
Estimated Reproduction (ER)	6474.7 (±588.4)NS	5608 (±1857.3)NS	6187.8 (±2778)NS	6644.2 (±3300.3)NS	4919.3(±1162.4)NS
Estimated Reproduction Inhibition (ERI)	0 (±9.1)NS	13.4 (±28.7)NS	4.4(±42.9)NS	-2.6 (±51)NS	24 (±18)NS

Letters indicate statistically significant differences ($p < 0.05$), and NS indicates no significant difference.
