# Supplementary material for: Efficacy of Entomopathogenic Staphylococcus Bacteria as a Biocontrol Agent against Rhipicephalus microplus Ticks: Assessing Reproductive Inhibition and Mortality Rates
Source: Microorganisms. 2024 Mar 11;12(3):551. doi: 10.3390/microorganisms12030551 (PMC10974063; doi:10.3390/microorganisms12030551)
Supplement: Supplementary file 1 [file microorganisms-12-00551-s001.zip › Table S4 corrected version.pdf]

Table S4. *S. succinus* (INIFAP 002-15) bioacaricidal activity of several variables obtained by modified AIT against the susceptible (Media Joya) *R. microplus strain.*

Variable	Control	1x106	1x107	1x108	1x109
Mortality (%)	0 (± 0)a	7.5 (± 5)ab	17.5 (± 9.6)b	15.0(± 5.8)ab	22.5 (± 12.6)b
Morbidity (%)	0 (± 0)a	15.0(± 5.8)ab	17.5 (± 9.6)ab	22.5 (±15)ab	32.5 (±18.9)b
Index of fertility (IF%)	36.1 (±1.7)NS	41.6 (±4.3)NS	40.9 (±4.2)NS	35.0(±4.4)NS	39.6 (±6.9)NS
Percentage inhibition of oviposition (%OI)	0 (±4.6)NS	-15.2 (±11.8)NS	-13.1 (±11.5)NS	3.11 (±12.3)NS	-9.7 (±19)NS
Hatching (H%)	65.6(±3.8)a	42.5 (±22.1)ab	53.2 (±6.9)ab	37.6 (±7.3)b	46.3 (±8.0)ab
Hatching Inhibition (HI%)	0(±5.8)a	35.6 (±33.4)ab	19.4 (±10.4)ab	43.1(±11)b	29.9(±12.2)ab
Estimated Reproduction (ER)	4738.7 (±235.9)NS	3617 (±2073.8)NS	4386.7(±974.4)NS	2638.3 (±647.3)NS	3712.4 (±1176.4)NS
Estimated Reproduction Inhibition (ERI)	0 (±5)NS	23.7 (±43.8)NS	7.4 (±20.6)NS	44.3 (±13.7)NS	21.6 (±24.8)NS

Letters indicate statistically significant differences ($p < 0.05$), and NS indicates no significant difference.
