# Supplementary material for: Efficacy of Entomopathogenic Staphylococcus Bacteria as a Biocontrol Agent against Rhipicephalus microplus Ticks: Assessing Reproductive Inhibition and Mortality Rates
Source: Microorganisms. 2024 Mar 11;12(3):551. doi: 10.3390/microorganisms12030551 (PMC10974063; doi:10.3390/microorganisms12030551)
Supplement: Supplementary file 1 [file microorganisms-12-00551-s001.zip › Table S5 corrected version.pdf]

Table S5. *S. xylosus* (INIFAP 009-16) bioacaricidal activity of several variables obtained by modified AIT against the susceptible (Media Joya) *R. microplus* strain.

Variable	Control	1x106	1x107	1x108	1x109
Mortality (%)	0 (±0)a	2.5 (±5)ab	7.5 (±5)ab	7.5 (±9.6)ab	15 (±5.8)b
Morbidity (%)	0 (± 0)a	10.0(± 8.2)ab	12.5 (± 5)ab	12.5 (± 5)ab	17.5 (±12.58)b
Index of fertility (IF%)	51.3 (± 3.7)a	48.9(± 4.4)ab	47.1 (± 1.4)ab	46.1 (± 5)ab	42.4 (±2.0)b
Percentage inhibition of oviposition (%OI)	0 (± 7.2)a	4.7 (± 8.5)ab	8.18 (± 2.8)ab	10.2 (±9.7)ab	17.4 (±3.9)b
Hatching (H%)	68.4(± 8.1)ab	71.9 (± 8.2)ab	55.7 (± 7.2)a	71.4(± 4.6)ab	75.3 (± 8.5)b
Hatching Inhibition (HI%)	0 (±10.11)ab	-5.08 (±12)ab	18.52 (±7.4)a	-4.39 (±4.6)ab	-10.1 (±12.5)b
Estimated Reproduction (ER)	7013.7 (±987.1)NS	7054(±1265)NS	5259 (±783.5)NS	6569.1 (±875)NS	6396.3(± 988)NS
Estimated Reproduction Inhibition (ERI)	0 (±14.1)NS	-0.58 (±18)NS	25(±11.2)NS	6.3 (±12.5)NS	8.8 (±14.1)NS

Letters indicate statistically significant differences ($p < 0.05$), and NS indicates no significant difference.
